# Decoding the bilateral vestibulopathy spectrum: etiology-based phenotypes, clinical profiles and pathways to implant candidacy

**DOI:** 10.3389/fneur.2026.1745221

**Published:** 2026-01-16

**Authors:** Joan Lorente-Piera, Raquel Manrique-Huarte, Ángel Ramos de Miguel, Ángel Ramos-Macías, Susana Benítez Robaina, Sebastián Picciafuoco, Manuel Manrique, Nicolás Pérez-Fernández

**Affiliations:** 1 Department of Otorhinolaryngology, Clínica Universidad de Navarra, Pamplona, Spain; 2 Cochlear Technology Centre Belgium, Mechelen, Belgium; 3 Department of Otorhinolaryngology, Complejo Hospitalario Universitario Insular MaternoInfantil, Las Palmas de Gran Canaria, Spain; 4 Department of Otorhinolaryngology, Clínica Universidad de Navarra, Madrid, Spain

**Keywords:** bilateral vestibulopathy, Ménière disease, vestibular implants, vestibular rehabilitation, vestibulotoxicity

## Abstract

**Introduction:**

Bilateral vestibulopathy (BVP) is a chronic, disabling disorder characterized by bilateral loss of vestibular function, leading to imbalance, oscillopsia, and falls. Despite established diagnostic criteria, clinical expression and rehabilitation potential vary widely across etiologies. Understanding these functional signatures is crucial for tailoring future neurostimulation strategies. The main objective of this study is to decode the etiological and functional heterogeneity of BVP and define clinical–functional profiles guiding vestibular or cochleo-vestibular implant candidacy.

**Methods:**

A multicenter retrospective study included 119 adults fulfilling Bárány Society criteria for definite BVP (>1 year). Comprehensive audiovestibular testing comprised pure-tone audiometry (PTA), video head impulse test (vHIT), vestibular evoked myogenic potentials (VEMPs), and dynamic posturography (SOT, LOS). Group comparisons used Kruskal–Wallis and Dunn–Bonferroni tests; oscillopsia predictors were analyzed by binary logistic regression.

**Results:**

Etiologies included idiopathic (29.41%), Ménière’s disease (26.89%), iatrogenic (18.49%), post-infectious (14.29%), cerebellar ataxia/CANVAS (6.72%), and post-traumatic (4.20%). Oscillopsia was reported by 48.74% and falls by 22.69%. Significant inter-etiological differences were found for semicircular canal gains (*p* < 0.001), with idiopathic and Ménière phenotypes showing higher vHIT gains and CANVAS/vestibulotoxic forms the lowest. IAAR-VEMP amplitudes were higher in idiopathic and Ménière groups than in CANVAS and vestibulotoxic etiologies (*p* < 0.01). Posturography differed across groups (SOT *p* = 0.007; LOS *p* = 0.010), CANVAS showing the poorest stability. Logistic regression identified reduced VOR gain in both the lateral and posterior semicircular canals as significant predictors of oscillopsia (LSC: right OR 0.185, *p* = 0.036; left OR 0.149, *p* = 0.035; PSC: right OR 0.167, *p* = 0.043; left OR 0.182, *p* = 0.047), together with an increased right PR index (OR 1.045, *p* = 0.010). Fourteen patients (11.76%) were qualified for cochleo-vestibular and the same amount for vestibular implant candidacy.

**Conclusion:**

BVP comprises etiology-specific phenotypes. Oscillopsia is driven mainly by VOR performance and PR index in LSC. Integrating auditory status, canal–otolith function, and compensation quality supports precision selection for vestibular versus cochleo-vestibular implantation.

## 1 Introduction

Bilateral vestibulopathy (BVP) is a chronic and disabling disorder characterized by bilateral reduction or loss of vestibular function, leading to persistent postural instability, impaired gaze stabilization, and substantial deterioration in quality of life. Although traditionally considered uncommon, recent population-based studies estimate a prevalence of 20–80 cases per 100,000 inhabitants, disproportionately affecting older adults (1). Unlike unilateral vestibular disorders, BVP lacks a consistent asymmetry and often presents without classical vertigo. Instead, patients typically report gait unsteadiness—particularly in darkness or on uneven surfaces—and blurred vision during head motion, reflecting bilateral vestibulo-ocular reflex (VOR) failure (2). These symptoms are associated with fear of falling, activity restriction, and psychosocial burden, making BVP one of the vestibular disorders with the greatest functional impact (3). Importantly, reliance on vestibular test abnormalities alone may underestimate disease burden, as patients can experience marked disability despite only partial objective deficits, underscoring the need for integrated clinical, functional, and patient-reported assessment (4).

BVP arises from a heterogeneous range of etiologies, including vestibulotoxicity, bilateral Ménière’s disease, meningitis, autoimmune inner-ear disorders, neurodegeneration, and trauma; nevertheless, up to half of cases remain idiopathic despite extensive diagnostic work-up (3, 5). Across etiologies, oscillopsia—the perception of environmental motion during head movement—emerges as a hallmark symptom, typically affecting 50–60% of patients. Its severity varies widely and depends not only on the degree of vestibular loss but also on compensatory mechanisms such as corrective saccades and multisensory substitution (6).

Auditory involvement is common in BVP, particularly in iatrogenic, post-infectious, and Ménière-related forms, with bilateral sensorineural hearing loss reported in up to 50–70% of patients (3–5). This overlap has major functional implications, as combined auditory and vestibular deficits exacerbate spatial disorientation and communication difficulties. On the other hand, patients with CANVAS or other genetic forms of BVP often retain useful hearing, whereas conditions such as DFNA9 are typically associated with significant cochlear involvement (5). However, despite hearing preservation, rehabilitation in CANVAS-related BVP is frequently more challenging due to the presence of cerebellar ataxia and sensory polyneuropathy, which limit central compensation mechanisms and reduce the effectiveness of vestibular rehabilitation (7). This contrast highlights the marked clinical and rehabilitative heterogeneity of BVP and underscores the need for etiology-aware therapeutic strategies.

Longitudinal studies indicate that spontaneous recovery of vestibular function is rare, and more than 80% of patients show no meaningful improvement over years of follow-up (8). Although vestibular rehabilitation remains the standard of care and can improve balance and subjective stability in selected cases, its efficacy is often limited in severe or near-complete vestibular loss, where peripheral input is minimal or absent (9–13). This therapeutic ceiling has driven the development of vestibular implants aimed at restoring artificial vestibular input through electrical stimulation. Early and more recent studies have demonstrated the feasibility of canal and otolithic stimulation, with encouraging effects on eye movements, postural control, and subjective stability in selected patients, including those receiving hybrid cochleo-vestibular devices (14–17).

Against this background, we hypothesize that etiology-specific functional signatures—integrating canal and otolith deficits, auditory status, saccadic reorganization, and graviceptive compensation—underlie much of the clinical heterogeneity observed in BVP and explain variability in symptoms such as oscillopsia beyond VOR gain alone. The present study therefore aims firstly, to characterize BVP heterogeneity by etiology across vestibular, auditory, and clinical dimensions; secondly, to describe the functional profiles of patients potentially eligible for vestibular or cochleo-vestibular implantation; and finally, to identify determinants of oscillopsia, testing whether dynamic gaze instability is more closely related to compensation quality and multisensory integration than to absolute vestibular loss, with direct implications for implant candidacy.

## 2 Methods

### 2.1 Study design

A retrospective, observational, and multicenter study was conducted including patients over 18 years of age diagnosed with BVP of more than 1 year of duration, who were recruited from two tertiary referral centers. The two hospitals were Clínica Universidad de Navarra (Pamplona and Madrid, Spain) and Hospital Universitario Insular Materno-Infantil de Gran Canaria (Las Palmas de Gran Canaria, Spain).

The study received ethical approval from the competent authorities and their respective local ethics committees. Written informed consent was obtained from all participants. The study was conducted in accordance with the ethical standards of the 1964 Declaration of Helsinki and its later amendments or comparable ethical standards, where applicable. All patients provided written informed consent prior to participation, and all procedures involving human participants adhered to the ethical standards of the institutional research committees.

### 2.2 Inclusion and exclusion criteria

*Inclusion criteria* contemplated patients over 18 years of age diagnosed with bilateral vestibulopathy of at least one year of evolution, according to the diagnostic criteria of the Bárány Society Consensus (1).

*Exclusion criteria* comprised patients with inner ear malformations or other structural abnormalities, unilateral vestibulopathy, or with significant psychiatric comorbidities likely to impair informed consent, data reliability, or adherence to the study protocol. Patients who refused or were unable to provide written informed consent or to participate in the study were also excluded.

### 2.3 Examination and complementary tests

All patients underwent a comprehensive physical examination, including otoscopy and a detailed otoneurological assessment using videonystagmography (VNG) (VideoFrenzel, Interacoustics VF505m, Assens, Denmark). The audiovestibular evaluation comprised pure-tone audiometry (PTA) (AC40, Interacoustics), vestibular evoked myogenic potentials (VEMP) (Eclipse, Interacoustics, Assens, Denmark), the video Head Impulse Test (vHIT) (ICS Impulse, GN Otometrics®, Natus Medical, Denmark) and dynamic posturography (EquiTest, NeuroCom International Inc., Clackamas, OR, USA). When clinically indicated, caloric testing (VisualEyes™, Interacoustics, Denmark) and dynamic visual acuity assessment (Balance Master System, NeuroCom International Inc., Clackamas, OR, USA) were also performed. All patients underwent audiological and vestibular testing on the same day of the diagnosis. All patients underwent audiological and vestibular testing on the same day of the diagnosis. For all audiovestibular analyses, data were evaluated and reported in an ear-specific manner. Comparisons were restricted to anatomically and physiologically equivalent measures only, that is, ipsilateral ear-to-ear comparisons across etiologies (right-to-right and left-to-left), and within the same test modality and end organ. No cross-lateral (right vs. left) or cross-parameter comparisons (e.g., between different semicircular canals or between cVEMP and oVEMP measures) were performed. This analytical approach was chosen to preserve clinical interpretability and to reflect real-world decision-making in implant candidacy.

- *Audiological evaluation*: Findings were reported in terms of pure tone average (PTA) thresholds from 0.25 to 4 kHz, expressed in decibels hearing level (dB HL).
- *Vestibular evaluation*

- *vHIT* was used to analyze the gain of the VOR, considering values below 0.6 as pathologic. As previously described by Rey-Martínez et al. (18), saccade clustering was analyzed using the open-source software HitCal for lateral semicircular canal, which classifies saccades into normal, scattered, or gathered categories according to the Pérez–Rey (PR) score. This score was calculated based on the temporal distribution and variability of saccadic responses. The time of occurrence of each group of saccades was recorded and categorized as the first, second, third, and fourth saccades. Only saccades with a minimum velocity of 65°/s were included in the analysis. For each group, the coefficient of variation (CV) of the peak velocities was calculated and labeled as CV1, CV2, CV3, and CV4, depending on their temporal order. The CV was defined as the ratio between the standard deviation and the mean, multiplied by 100 to express it as a percentage when required. The global PR score was then derived using the following formula:
PR=2.5×(0.8×CV1+0.2×CV2 ).
  According to the authors’ description, PR values range from 0, indicating maximally gathered saccades where all occur simultaneously, to 100, representing maximally scattered responses (18).
- For *VEMP*, both cervical (cVEMP) and ocular (oVEMP) tests were conducted using both air-conducted and bone-conducted stimulation. An abnormal vestibular function was defined as bilateral absence of the response or a VEMP response in both ears with an interaural asymmetry ratio (IAAR %) exceeding 40% (19). Burst tones of 500 Hz were used for monaural auditory stimulation using previously calibrated hearing aids. The intensity of the acoustic stimulus was 97 dB normalized hearing level. A Blackman envelope was configured (rise/fall time 2 ms, plateau time 0 ms). 100 averages were presented at a rate of 5.1/s. The response evoked by cVEMP describes a positive (p13) and negative (n23) wave. In oVEMP, the response presents a negative (n10) and positive (p16) wave.
- For *dynamic posturography* and for the purposes of this study, only the global composite score of the Sensory Organization Test (SOT) and the Limits of Stability (LOS) were considered. Normal composite SOT values were defined as a score of ≥69, indicating preserved postural control, while normal LOS values were based on age-standardized parameters, with ≥64 cm^2^ generally considered within normal limits according to the manufacturer’s normative database (NeuroCom International Inc., Clackamas, OR, USA) and expressed as age-standardized composite scores. Both tests were performed under standardized conditions, and all recordings were analyzed according to the established normative data by Cevette et al. (20) in 1995.
- The *caloric test* was performed using water irrigation at three standard temperatures (44 °C, 30 °C, and ice water at approximately 4 °C), with each ear tested separately. This evaluation was only conducted in patients with a clinical suspicion of bilateral vestibulopathy but with horizontal canal vHIT gains greater than 0.6. The responses were analyzed based on the slow-phase velocity (SPV) of the induced nystagmus (21).
- *Dynamic visual acuity* was only evaluated in those patients referring oscillopsia. Participants were positioned 1.5 m from the visual display while fixating on optotypes projected at eye level. Static visual acuity was first recorded, followed by dynamic measurements during active horizontal head rotations at controlled peak velocities of 100–130°/s and amplitudes of 20–30° to each side. The difference between static and dynamic visual acuity, expressed in logMAR units, was calculated separately for rightward and leftward rotations. Results were considered within normal limits when the dynamic–static difference was <0.7 logMAR, corresponding to effective VOR compensation and retinal slip <70 ms.

### 2.4 Clinical and demographic evaluation

Clinical and demographic data were collected for all participants, including sex, age at diagnosis, etiology and disease evolution. Functional symptoms were systematically documented, including the presence or absence of imbalance, oscillopsia, vertigo spells during last year, and falls during daily activities. This information was used to characterize the clinical profile of each patient and to explore potential associations between etiology, disease evolution, and symptom in subjects potentially eligible for vestibular implantation.

### 2.5 Implant candidacy selection. Inclusion, exclusion criteria and the implant selected

Eligible participants were adults (≥18 years) with a diagnosis of bilateral vestibulopathy following the Bárány Society research criteria (1) of at least one year’s duration. The implanted ear had to show absent cVEMP and oVEMP responses and demonstrate optimal anatomical conditions for electrode insertion on CT scans and MRI (patent vestibule and intact vestibular nerve). Participants also needed to be able to undergo surgery and comply with a personalized rehabilitation program. From an auditory standpoint, for the vestibular-only device, candidates were considered those presenting a moderate to severe hearing loss (PTA 40–70 dB) in the ear to be implanted. For the combined cochleo-vestibular device, eligibility required a severe-to-profound hearing loss in the implanted ear and a speech recognition score below 50% at 65 dB on the Cárdenas and Marrero (22) disyllabic word test, measured under best hearing-aided conditions.

Exclusion criteria for implantation, even in patients fulfilling functional audiovestibular criteria, included inner ear ossification or malformations preventing full electrode insertion, middle ear disease or conductive hearing loss, retrocochlear pathology, and central causes of hearing or balance impairment. Central nervous system conditions comprised cerebellar ataxias as CANVAS, Parkinson’s disease, brainstem or cerebellar lesions, and other vascular or neurodegenerative disorders affecting gait or oculomotor control, as well as downbeat nystagmus syndrome, all identified through detailed neuro-otological and neurological examination and neuroimaging when appropriate. Patients with persistent postural–perceptual dizziness (PPPD), visually induced or functional vertigo, oculomotor abnormalities, peripheral neuropathies, unilateral vestibular deficits, or intoxication due to alcohol or drugs were also excluded, given the central or non-vestibular contribution to symptoms. Additional exclusion criteria included chronic depression, dementia, or other cognitive disorders likely to interfere with correct device use or outcome assessment; active psychiatric disease; poor compliance or unrealistic expectations regarding potential benefit; pregnancy or breastfeeding; participation in other clinical trials within the previous 30 days; or explicit patient refusal to participate.

When referring to the cochleo-vestibular implant used in this study, we employed the BionicVEST 1 (Cochlear Ltd., Sydney, Australia) model, which consists of two main components: an internal and an external unit. The internal component comprises a receiving coil, an intracochlear electrode array with 19 contacts configured according to the CI512 Contour Advanced (Cochlear Ltd., Sydney, Australia) perimodiolar design, a vestibular electrode array with three full-band contacts spaced 0.2 mm apart, and a reference ball electrode. In contrast, the vestibular-only implant, called BIONIC-VEST 2 (Cochlear, Sydney, Australia), consists solely of the vestibular electrode array and the reference electrode, without the intracochlear array.

The external component of both corresponds to the commercially available Nucleus 6 or 7 sound processor, which is conventionally positioned beneath the periosteum and oriented toward the zygomatic arch.

Stimulation parameters consisted of generating balanced biphasic pulse trains. The pulse width was set to 25 μs, with a stimulation frequency of 900 pulses per second. Monopolar stimulation was applied using the ball and plate electrodes as reference (MP1+2 configuration). The modulation of stimulation varied according to the target structure: for cochlear stimulation, the amplitude of the stimulus was modulated between the threshold (T) and comfort (C) levels, defining the dynamic range, whereas otolithic stimulation was delivered at a constant current level.

### 2.6 Statistical analysis

Continuous variables were tested for normality using the Shapiro–Wilk test. Normally distributed data are presented as mean ± standard deviation (SD), whereas non-normally distributed variables, such as PR indices, are reported as median [interquartile range, IQR]. As most continuous parameters did not meet normality assumptions, comparisons across etiological groups and implant-candidacy categories were performed using the Kruskal–Wallis test. Pairwise comparisons were conducted using Dunn’s *post hoc* test with Bonferroni adjustment; therefore, *p*-values displayed for *post hoc* pairwise comparisons are adjusted for multiple testing. Categorical variables and symptom frequencies were analyzed using the Chi-square test or Fisher’s exact test, as appropriate. Again, when multiple categorical comparisons were performed, Bonferroni correction was applied, and the reported *p*-values for these multiple comparisons are adjusted.

The association between oscillopsia and vestibular or functional parameters was assessed using binary univariate logistic regression, with oscillopsia (present/absent) as the dependent variable. Semicircular canal VOR gains (SSC, LSC, and PSC), PR indices, posturographic performance (SOT), visual acuity, age, and disease duration were each evaluated as independent predictors in separate models. Results are expressed as odds ratios (OR) with 95% confidence intervals (CI), and statistical significance was assessed at a two-sided alpha level of 0.05.

All statistical analyses were performed using GraphPad Prism v8.0.1 (GraphPad Software Inc., San Diego, CA, USA).

## 3 Results

### 3.1 Demographic and etiological distribution

Of the 170 patients initially diagnosed with BVP, 51 (30%) were classified as probable cases and 119 (70%) fulfilled the Bárány Society criteria for definite BVP (1), constituting the final study cohort. The demographic characteristics of the patients included in the study are summarized in Table 1.

**Table 1:** Summary of demographic data and clinical characteristics of the patients included in the study.

Demographic and clinical data	
Patients with definite BVP (men: women)	64 (53.78%)/55 (46.22%) patients
Age (mean ± SD)	66.67 ± 10.18 (22–98) years
Disease duration (mean ± SD)	9.47 ± 12.68 (1–78) years
Etiology	
Idiopathic	35 (29.41%) patients
Ménière disease	32 (26.89%) patients
Iatrogenic	22 (18.49%) patients
Post-infectious	17 (14.29%) patients
Cerebellar ataxia/CANVAS	8 (6.72%) patients
Post-traumatic	5 (4.20%) patients

All patients (*n* = 119, 100%) presented with chronic imbalance, fulfilling the diagnostic criteria for BVP, while oscillopsia—a hallmark symptom of the disease—was reported by 58 participants (48.74%). Additionally, 66 patients (55.46%) experienced episodic vertigo and 27 (22.69%) had a documented history of falls.

### 3.2 Overall audiovestibular findings

From a general perspective, the audiometric evaluation revealed slightly poorer hearing thresholds in the right ear. Although the right ear exhibited a higher mean PTA, both showed a similar distribution of severe-to-profound hearing loss range: 32 patients (26.89%) on the right ear and 30 patients (25.21%) on the left ear.

vHIT results showed that in general, the lowest gains were observed in the posterior semicircular canal (PSC), followed by the lateral semicircular canal (LSC) and superior semicircular canal (SSC). Notably, 18 patients (15.13%) who exhibited LSC gain values above 0.6 but presented a clinical presentation consistent with BVP, showed complete vestibular areflexia following ice–water irrigation in both ears in the caloric test, with a mean SPV of 0.42 ± 0.54°/s. Finally, an intermediate value was observed in both ears for the PR index, which did not differ statistically.

cVEMPs were absent in 60 patients (50.42%), while oVEMPs were absent in 63 patients (52.94%). Finally, posturography outcomes based on the SOT and LOS, as well as dynamic visual acuity were consistently found within pathological ranges. A more detailed description of these results is provided in Supplementary Table, in which no statistically significant differences were observed in any variable when compared with the contralateral side.

### 3.3 Clinical and audiovestibular results according to etiology

Symptom analysis across etiological groups revealed distinct clinical patterns. While unsteadiness was a universal finding among all participants, the distribution of other symptoms varied notably between etiologies. Vertigo episodes were most frequent in patients with Ménière’s disease, consistent with its episodic course, whereas oscillopsia was predominantly reported in subjects with vestibulotoxic, post-infectious, and cerebellar or ataxic origins, reflecting a more chronic vestibular impairment. Conversely, the occurrence of falls was disproportionately higher in individuals with cerebellar ataxia or CANVAS, suggesting a more severe impact on postural control in this subgroup. Overall, these results emphasize the heterogeneous clinical presentation of bilateral vestibulopathy, where symptom burden and compensation profiles differ depending on the underlying cause. It can be inferred in Table 2.

**Table 2:** Distribution of symptoms according to etiology.

Symptom	Etiology	Result	*p* value
Unsteadiness (*n* = 119)	Idiopathic	35 (100%)	– (Constant across groups)
	Iatrogenic	32 (100%)	
	Post-infectious	22 (100%)	
	Ménière disease	17 (100%)	
	Cerebellar ataxia/CANVAS	8 (100%)	
	Post-traumatic	5 (100%)	
Oscillopsia (*n* = 58)	Idiopathic	9 (25.71%)	0.028*
	Iatrogenic	17 (77.27%)	
	Post-infectious	10 (58.82%)	
	Ménière disease	13 (47.06%)	
	Cerebellar ataxia/CANVAS	7 (87.50%)	
	Post-traumatic	2 (40.00%)	
Vertigo spells last year (*n* = 66)	Idiopathic	14 (40.00%)	0.001**
	Iatrogenic	11 (50.00%)	
	Post-infectious	7 (41.18%)	
	Ménière disease	29 (90.62%)	
	Cerebellar ataxia/CANVAS	3 (37.50%)	
	Post-traumatic	2 (40.00%)	
Risk of falls (*n* = 27)	Idiopathic	6 (17.14%)	<0.001***
	Iatrogenic	5 (22.73%)	
	Post-infectious	3 (17.65%)	
	Ménière disease	5 (15.62%)	
	Cerebellar ataxia/CANVAS	6 (75.00%)	
	Post-traumatic	2 (40.00%)	

*p < 0.05, **p < 0.01, ***p < 0.001.

**Figure 1:**
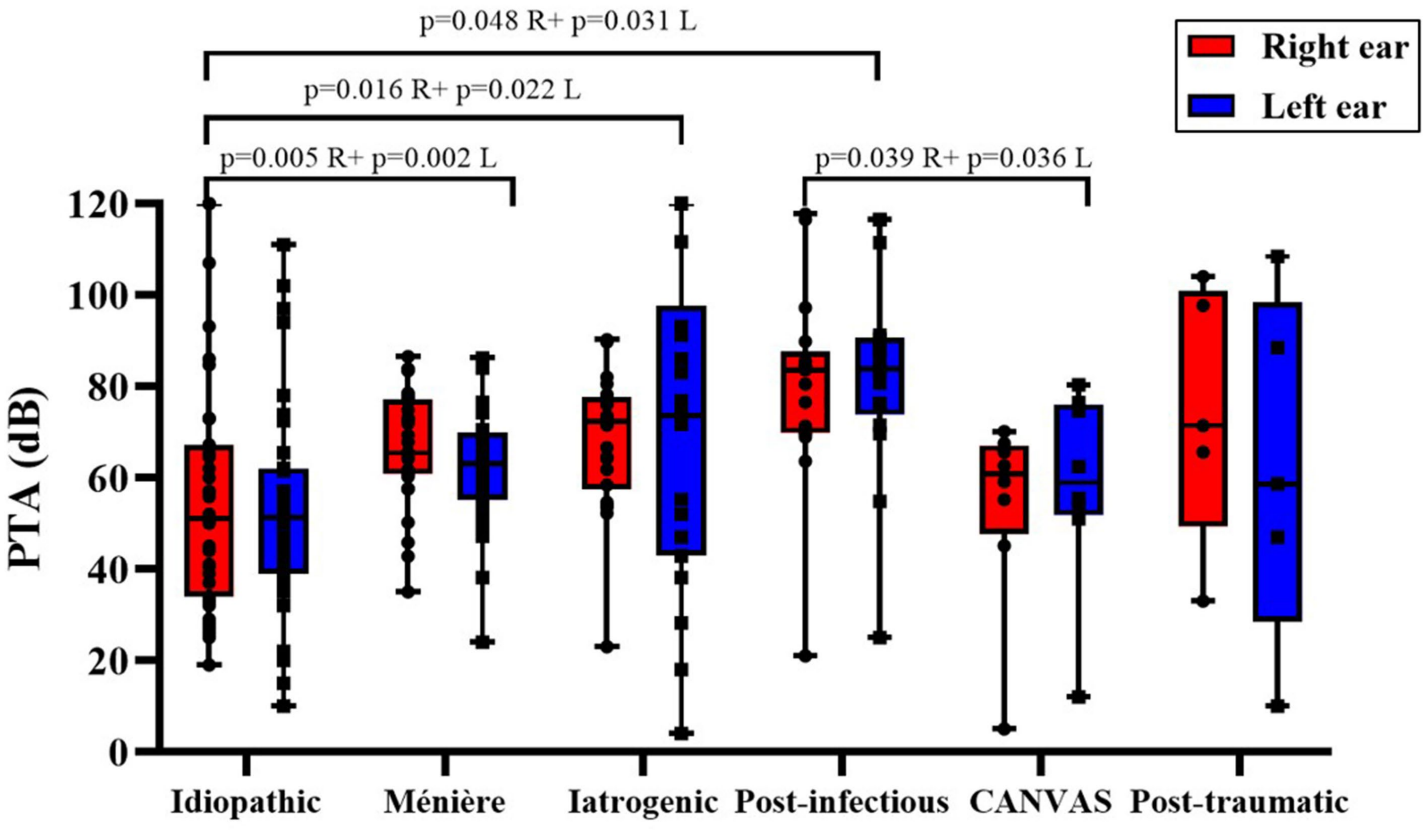
Audiometric results (PTA, dB) for the right and left ears across etiological groups. Boxes represent the median and interquartile range (IQR); whiskers indicate the minimum and maximum values; individual datapoints are shown. Pairwise comparisons between etiologies were performed ipsilaterally (right-to-right and left-to-left), and exact *p*-values are reported above brackets for each side. R, Right ear; L, Left ear.

As illustrated in Figure 1, despite the absence of a generalized difference across groups in the Kruskal–Wallis (Table 3), mean hearing thresholds were broadly comparable among etiologies. The post-infectious subgroup exhibited the highest PTA values, whereas idiopathic and especially CANVAS/ataxia patients tended to show lower thresholds. Considerable variability was observed in the idiopathic and iatrogenic groups, as reflected by the wide dispersion of their boxplots. Overall, both ears demonstrated similar distributions of hearing loss severity, predominantly within the moderate-to-severe range.

**Table 3:** Mean/median audiovestibular test results distributed by etiology.

Etiology	PTA (dB)		vHIT								IAAR VEMP (%)		Posturography	
	Right ear	Left ear	Right SSC	Right LSC	Right PSC	Left SSC	Left LSC	Left PSC	Right PR	Left PR	cVEMP	oVEMP	SOT (score)	LOS (cm^2^)
Idiopathic	51.28	45.69	0.71	0.54	0.57	0.64	0.51	0.58	66.72	58.22	53.90	49.36	52.18	61.42
Ménière	71.36	71.49	0.59	0.53	0.54	0.57	0.52	0.50	49.52	46.70	50.48	52.27	49.76	53.19
Iatrogenic	70.71	71.36	0.40	0.31	0.28	0.31	0.29	0.27	54.16	44.40	36.82	31.12	46.37	49.08
Post-Infectious	84.83	82.67	0.41	0.39	0.37	0.39	0.36	0.34	48.55	50.22	38.06	31.75	36.94	53.19
CANVAS	64.83	58.67	0.32	0.20	0.19	0.39	0.19	0.21	55.67	79.00	20.63	18.78	33.38	48.75
Post-traumatic	74.33	69.83	0.59	0.55	0.53	0.57	0.56	0.55	26.32	29.84	22.33	20.00	55.67	55.33
*p* value	0.111	0.090	<0.001***			<0.001***			0.147	0.099	0.002**	<0.001***	0.007**	0.010*

*p < 0.05, **p < 0.01, ***p < 0.001.

**Figure 2:**
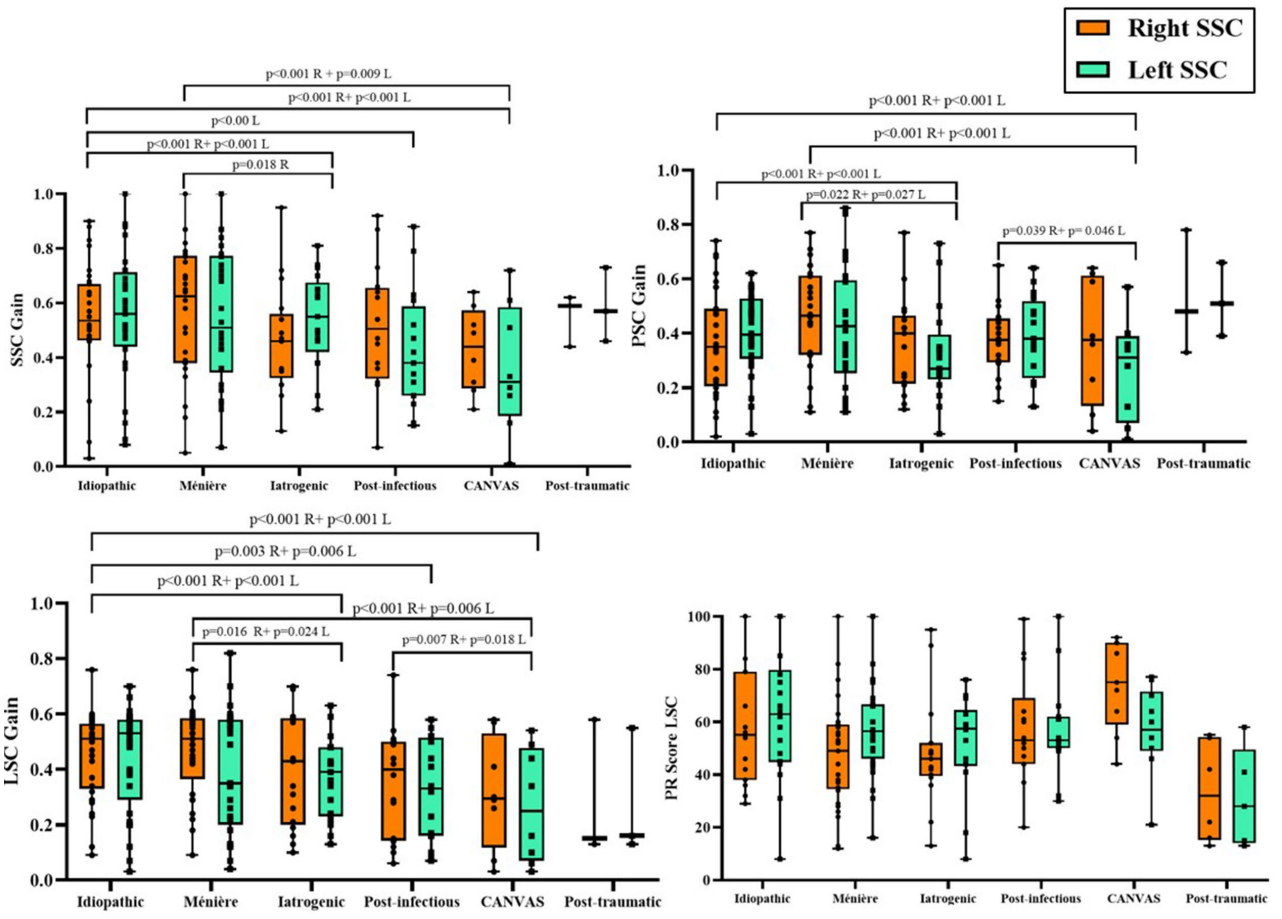
vHIT results according to etiology, including semicircular canal VOR gains (SSC, LSC, PSC) and saccadic reorganization assessed by the PR index. Right and left sides are displayed separately, and all comparisons were performed ipsilaterally (right-to-right and left-to-left across etiologies), with no cross-lateral comparisons. Boxes represent the median and interquartile range (IQR); whiskers indicate the minimum and maximum values; individual datapoints are shown. Exact *p*-values for statistically significant pairwise comparisons are reported above brackets. R, Right; L, Left.

As illustrated in Figure 2 and summarized in Table 3, significant inter-etiological differences were identified in VOR gain across all semicircular canals (*p* < 0.001). Idiopathic patients showed the highest gains, followed by those with post-traumatic lesions and Ménière’s disease, whereas CANVAS and vestibulotoxic etiologies presented the most pronounced deficits. Post-infectious cases occupied an intermediate position.

The LSC showed the largest intergroup separation, confirming a consistent pattern of functional decline across canal planes. Bilateral symmetry of results further supports the uniformity of vestibular damage within each etiology. Even more, in LSC, despite PR values did not differ significantly between groups (*p* > 0.05), again CANVAS and vestibulotoxic etiologies presented the most pronounced deficits in terms of a higher level of saccades disorganization with higher scores in the index.

**Figure 3:**
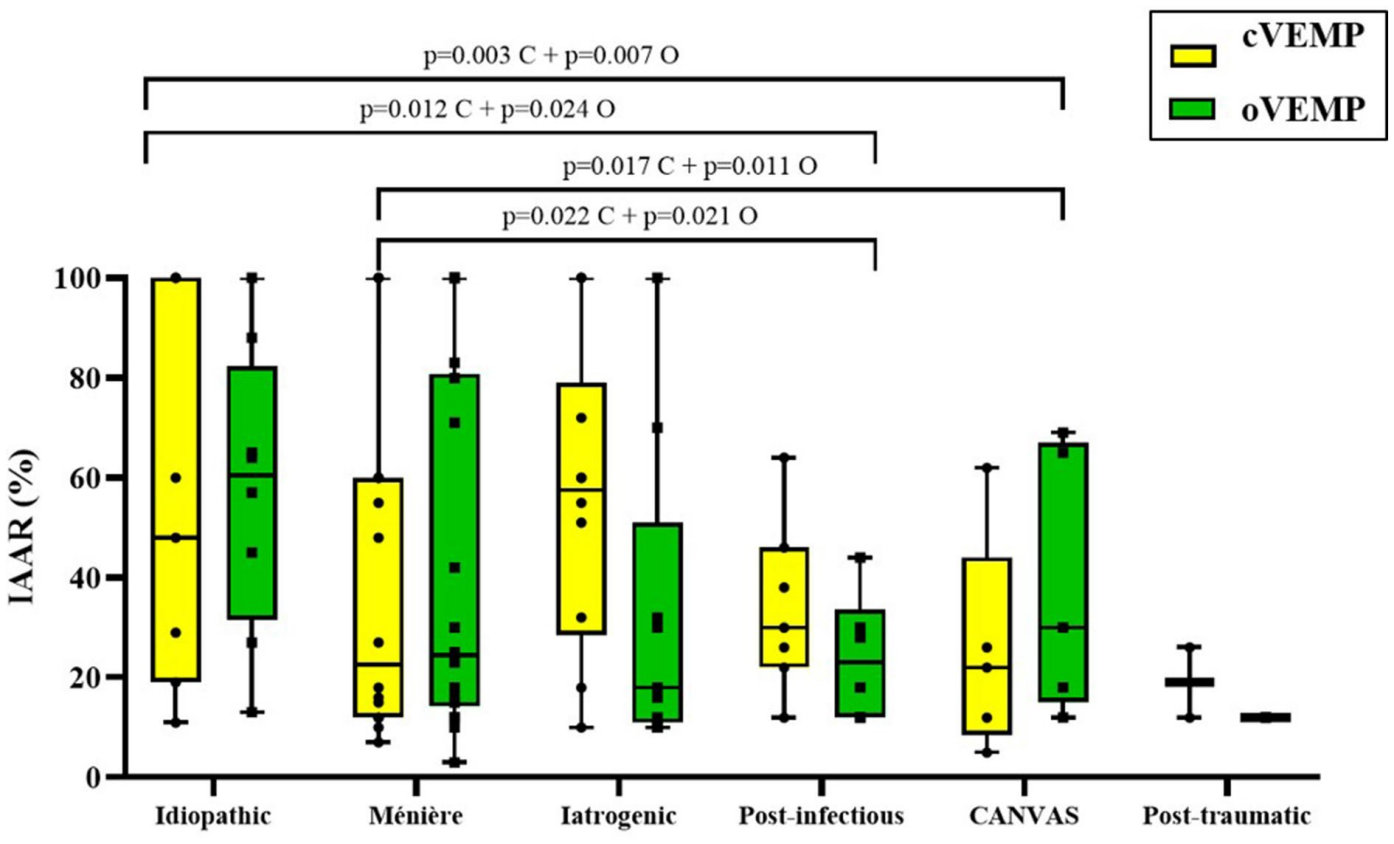
Otolith function assessed by IAAR (%) from cVEMP and oVEMP responses according to etiology. cVEMP and oVEMP results are displayed separately by modality, and comparisons were performed only within the same modality across etiologies, avoiding cross-modality comparisons. Boxes represent the median and interquartile range (IQR); whiskers indicate the minimum and maximum values; individual data points are shown. Exact *p*-values for statistically significant pairwise comparisons are reported above brackets. C, cervical; O, ocular.

Regarding VEMPS, the rate of absent responses differed markedly across etiologies: CANVAS (cVEMP 75.00%, oVEMP 87.50%) and post-traumatic (60.00, 80.00%) exhibited the highest absence rates, followed by iatrogenic/vestibulotoxic (63.63, 59.09%). In contrast, absence rates were lower in idiopathic (45.71, 42.86%), Ménière’s disease (40.62, 37.50%), and post-infectious (47.06, 41.17%) patients. Among participants with measurable responses, as shown in Table 3, Figure 3, IAAR values revealed a consistent functional hierarchy: idiopathic and Ménière’s disease exhibited the highest amplitudes, while CANVAS, vestibulotoxic, and post-traumatic etiologies displayed markedly lower IAAR values. Post-hoc comparisons confirmed that idiopathic and Ménière’s patients had statistically significant higher IAAR than CANVAS and vestibulotoxic groups for both cervical and ocular recordings, despite presenting a clearly lower percentage of absent responses.

**Figure 4:**
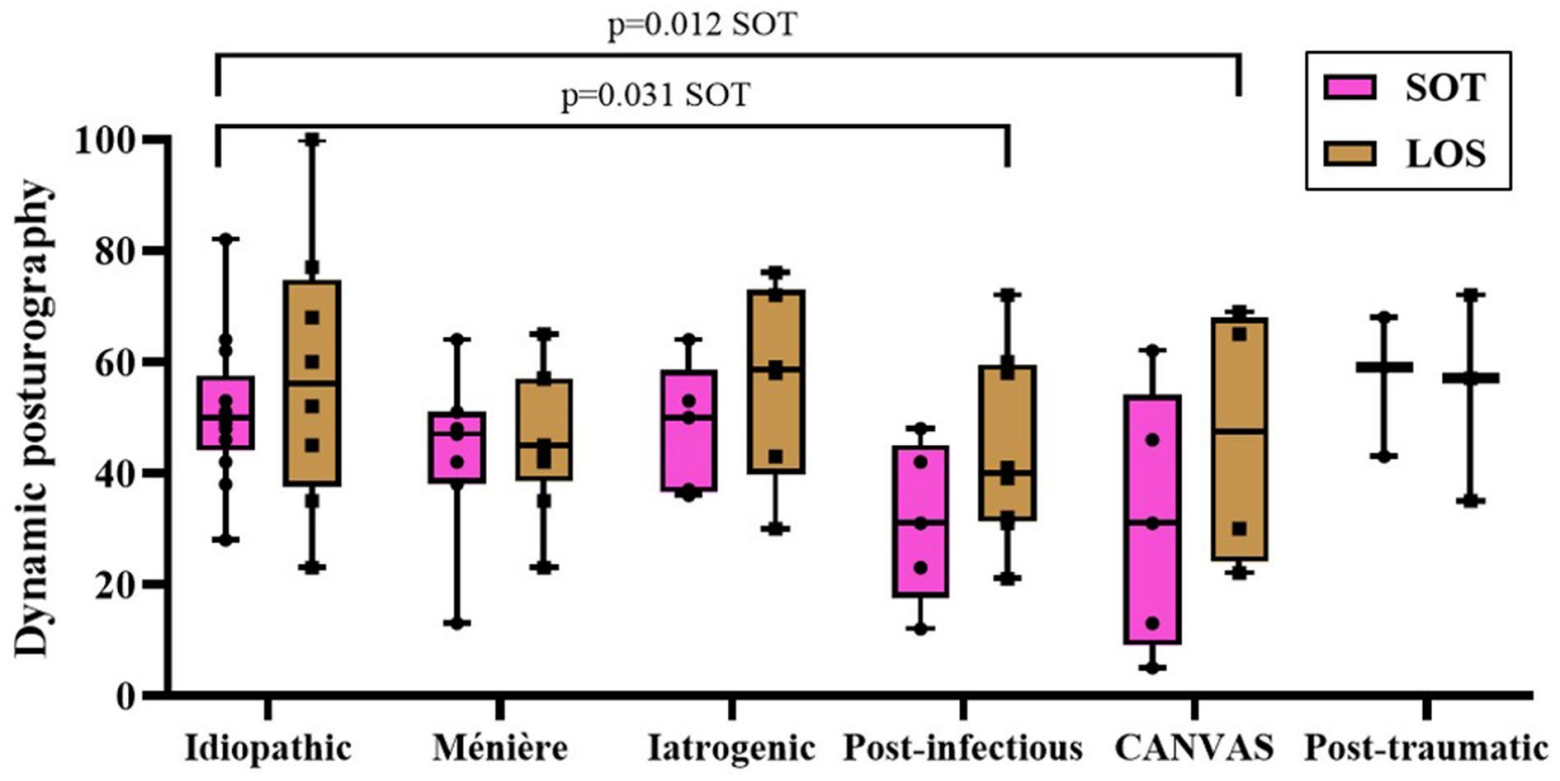
Dynamic posturography performance according to etiology, assessed by SOT and LOS scores. Comparisons were performed between etiological groups within each posturographic parameter. Boxes represent the median and interquartile range (IQR); whiskers indicate the minimum and maximum values; individual datapoints are shown. Exact *p*-values for statistically significant pairwise comparisons are reported above brackets.

Finally, as shown in Figure 4 and summarized in Table 3, dynamic posturography revealed also significant inter-etiological differences for both SOT and LOS (*p* = 0.007 and *p* = 0.010, respectively). Idiopathic, post-traumatic and Ménière’s disease patients achieved the highest scores, indicating better postural stability, whereas post-infectious and CANVAS groups showed the lowest performance. Iatrogenic etiologies occupied an intermediate position, with broad variability and without clear pairwise separation.

### 3.4 Variables involved in the development of oscillopsia

Binary univariate logistic regression analyses identified several vestibular predictors significantly associated with the presence of oscillopsia (Table 4). Reduced VOR gain in the LSC was significantly associated with oscillopsia on both sides (right LSC: OR 0.185, *p* = 0.036; left LSC: OR 0.149, *p* = 0.035). Likewise, reduced PSC gain was also significantly associated with oscillopsia bilaterally (right PSC: OR 0.167, *p* = 0.043; left PSC: OR 0.182, *p* = 0.047).

**Table 4:** Univariate logistic regression analysis of predictors associated with the presence of oscillopsia.

Predictor	Coefficient	Odds ratio	CI (95%)	*p* value
Age	0.002	1.002	0.964–1.040	0.201
BVP evolution	0.020	1.020	0.955–1.090	0.949
Right gain SSC	−3.004	0.077	0.001–5.980	0.510
Right gain LSC	−1.687	0.185	0.088–0.999	0.036*
Right gain PSC	−1.892	0.167	0.090–0.995	0.043*
Left gain SSC	−2.967	0.068	0.001–4.630	0.455
Left gain LSC	−1.908	0.149	0.050–0.991	0.035*
Left gain PSC	−2.233	0.182	0.012–0.968	0.047*
PR Right LSC	0.044	1.045	1.016–1.075	0.010*
PR Left LSC	0.009	1.009	0.985–1.033	0.054
Caloric test (SPV)	−0.008	0.986	0.959–1.038	0.714
SOT score	0.050	1.052	0.982–1.126	0.227
Visual acuity	−0.006	0.994	0.946–1.044	0.843

*p < 0.05, **p < 0.01, ***p < 0.001.

In addition, the PR index of the right LSC emerged as a significant positive predictor of oscillopsia (OR 1.045, *p* = 0.010), whereas the left PR index showed only a trend toward significance (OR 1.009, *p* = 0.054). In contrast, SSC gains, SPV results of the caloric test, posturographic performance (SOT score), visual acuity and clinical variables as age, disease duration, were not significantly associated with oscillopsia (*p* > 0.05 for all).

### 3.5 Clinical and audiovestibular results according to implant candidacy

Of the 119 patients included in the study, those who failed to achieve sufficient improvement after conventional vestibular rehabilitation were evaluated for potential implantable therapies. After excluding patients with central or neurodegenerative disorders—such as CANVAS—from implant eligibility, 28 patients (23.53%) were considered suitable candidates for implantable devices: 14 (11.76%) for a cochleo-vestibular implant (Bionic-VEST 1) and 14 (11.76%) for a vestibular implant (Bionic-VEST 2). The remaining 91 patients (76.47%) were classified as non-candidates. Group characteristics according to etiology and clinical features are summarized in Table 5.

**Table 5:** Distribution of symptoms according to implant candidacy.

Candidacy	Etiology	Result	Symptom	Result
Non candidates (*n* = 91)	Idiopathic	26 (28.57%)		
	Iatrogenic	18 (19.78%)	Unsteadiness	91 (100%)
	Post-infectious	12 (13.19%)	Oscillopsia	44 (48.35%)
	Ménière disease	22 (24.18%)	Vertigo spells	57 (60.63%)
	CANVAS	8 (8.79%)	Falls	15 (16.48%)
	Post-traumatic	5 (5.49%)		
Cochleo-vestibular implant candidates (*n* = 14)	Idiopathic	3 (21.42%)		
	Iatrogenic	2 (14.28%)	Unsteadiness	14 (100%)
	Post-infectious	3 (21.42%)	Oscillopsia	8 (57.14%)
	Ménière disease	6 (42.86%)	Vertigo spells	5 (35.71%)
			Falls	7 (50.00%)
Vestibular implant candidates (*n* = 14)	Idiopathic	6 (42.86%)		
	Iatrogenic	2 (14.28%)	Unsteadiness	14 (100%)
	Post-infectious	2 (14.28%)	Oscillopsia	6 (42.85%)
	Ménière disease	4 (28.57%)	Vertigo spells	4 (28.57%)
			Falls	5 (35.71%)
*p* values	Idiopathic	0.589		
	Iatrogenic	0.303	Unsteadiness	–(Constant)
	Post-infectious	0.247	Oscillopsia	0.094
	Ménière disease	0.412	Vertigo spells	0.866
	CANVAS	0.332	Falls	0.821
	Post-traumatic	0.479		

Among cochleo-vestibular implant candidates, Ménière’s disease was the most frequent etiology, followed by idiopathic and post-infectious causes. In contrast, idiopathic and Ménière’s disease predominated among vestibular implant candidates, with iatrogenic and post-infectious etiologies less commonly represented. Post-traumatic cases were infrequent across implant groups.

Unsteadiness was present in all patients regardless of candidacy status. Oscillopsia was more frequently observed among implant candidates—particularly in the cochleo-vestibular group—compared with non-candidates, showing a trend toward statistical significance (*p* = 0.094). Falls were more common among cochleo-vestibular implant candidates, whereas vertigo spells were predominantly reported in non-candidates.

**Figure 5:**
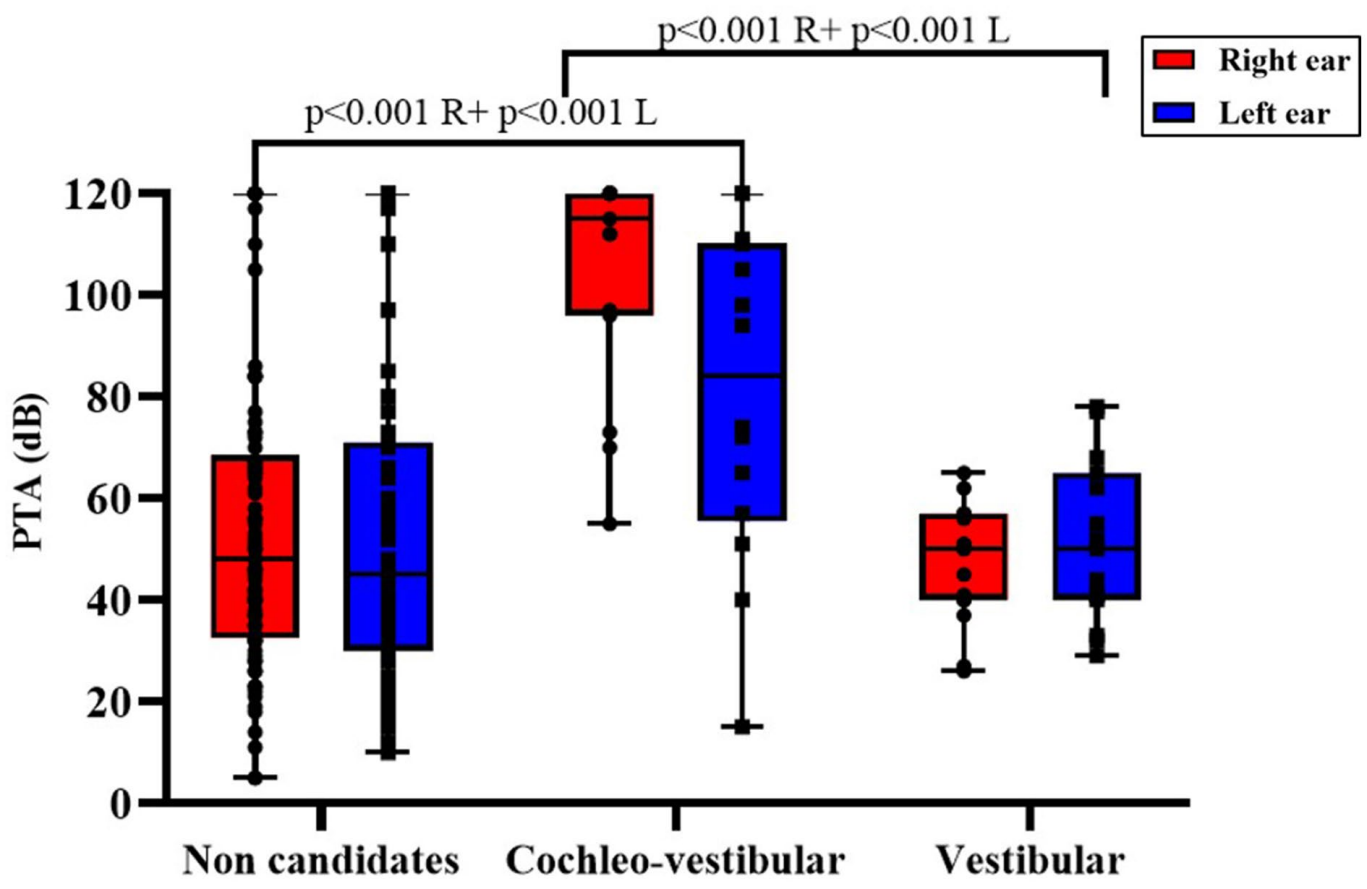
Audiometric results (PTA, dB) for the right and left ears according to implant candidacy. Comparisons were performed ipsilaterally (right-to-right and left-to-left) between candidacy groups. Boxes represent the median and interquartile range (IQR); whiskers indicate the minimum and maximum values; individual datapoints are shown. Exact *p-values for statistically significant p*airwise comparisons are reported above brackets. R, Right ear; L, Left ear.

From an audiometric perspective, significant intergroup differences were observed (*p* < 0.001). As expected, patients selected for the cochleo-vestibular implant exhibited markedly poorer hearing thresholds in both ears (right PTA 104.64 dB, left PTA 89.06 dB), consistent with the combined auditory and vestibular indication of this device. In contrast, candidates for the vestibular implant showed relatively preserved cochlear function, with mean PTA values (46.91 dB right, 47.78 dB left) comparable to those of non-candidates (56.38 dB right, 55.11 dB left). These findings are summarized in Table 6, Figure 5.

**Table 6:** Mean/median audiovestibular test results distributed by implant candidacy groups.

Candidacy	PTA (dB)		vHIT								Posturography	
	Right ear	Left ear	Right SSC	Right LSC	Right PSC	Left SSC	Left LSC	Left PSC	Right PR	Left PR	SOT (score)	LOS (cm^2^)
Non candidate	56.38	55.11	0.61	0.49	0.43	0.59	0.51	0.46	57.32	56.05	45.29	48.16
Cochleo-vestibular candidate	104.64	89.06	0.51	0.43	0.32	0.45	0.40	0.34	78.64	80.56	39.18	57.39
Vestibular candidate	46.91	47.78	0.56	0.48	0.41	0.53	0.47	0.43	63.81	64.08	47.13	53.24
*p* value	<0.001***		0.017*	0.044*	0.031*	0.014*	0.009**	0.042*	<0.001***		0.424	0.709

*p < 0.05, **p < 0.01, ***p < 0.001.

**Figure 6:**
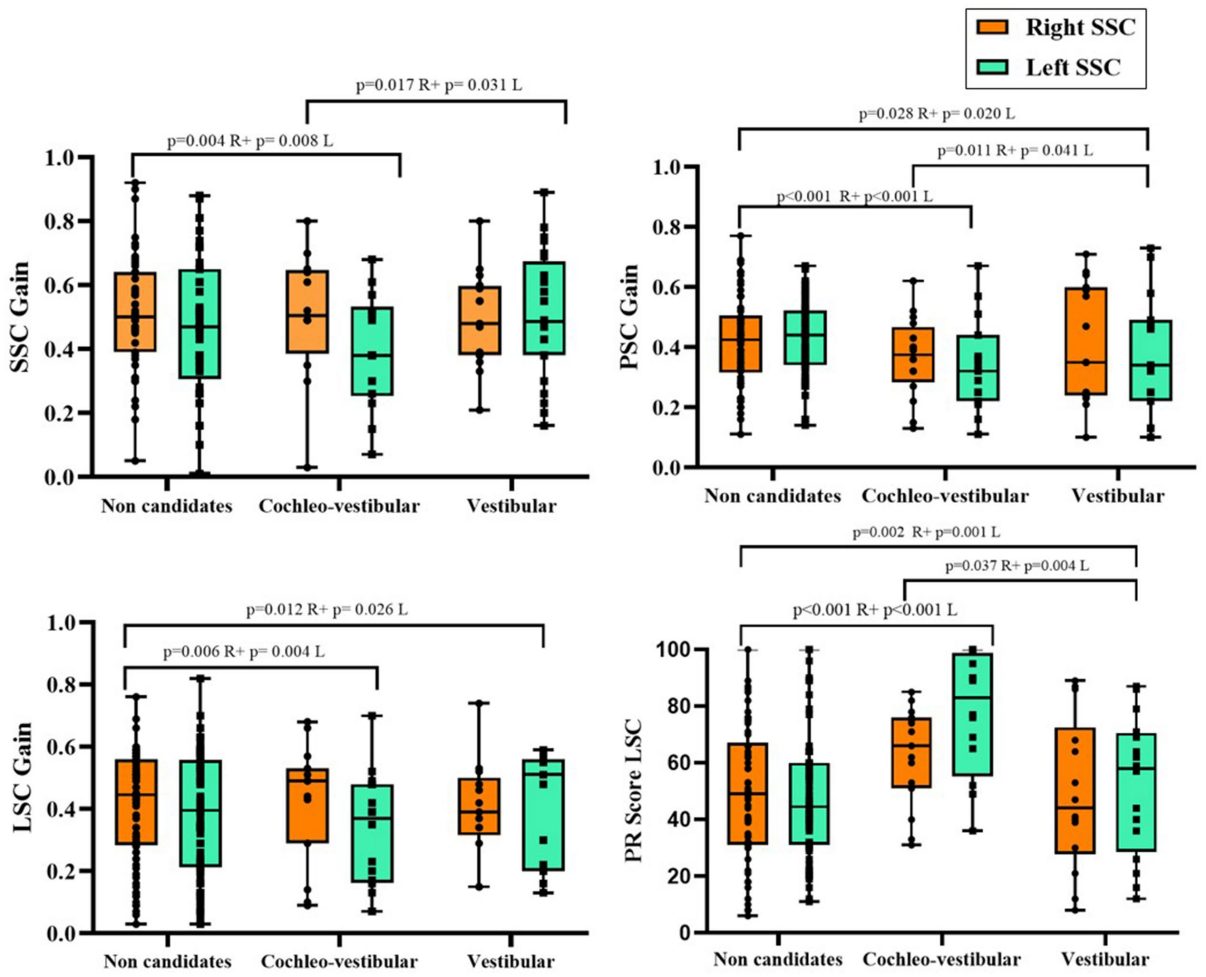
vHIT results according to implant candidacy, including semicircular canal VOR gains (SSC, LSC, PSC) and saccadic reorganization assessed by the PR index. Right and left sides are displayed separately, and all comparisons were performed ipsilaterally (right-to-right and left-to-left) between candidacy groups. Boxes represent the median and interquartile range (IQR); whiskers indicate the minimum and maximum values; individual data points are shown. Exact *p*-values for statistically significant pairwise comparisons are reported above brackets. R, Right; L, Left.

Global vHIT analysis revealed significant intergroup differences across all semicircular canal planes (Table 6, Figure 6). The cochleo-vestibular candidate group demonstrated the most pronounced impairment, with significantly reduced gains in all canals and markedly elevated PR indices, indicating severe bilateral vestibular dysfunction with substantial saccadic disorganization. The vestibular implant candidate group showed slightly higher, yet still pathological, canal gains, suggesting partial residual function or compensatory mechanisms. Non-candidates exhibited comparatively higher gains and lower PR values, consistent with milder vestibular impairment. Overall, Kruskal–Wallis testing with *post-hoc* comparisons confirmed significant differences between groups for all canal gains (*p* < 0.05). In addition, among the 18 patients who underwent caloric testing, 16 were classified as non-candidates for implantation, with a mean SPV of 0.39 ± 0.54°/s. Of the remaining two patients, one, with an SPV of 1.10°/s, was considered a candidate for a cochleo-vestibular implant, while the other, with an SPV of 0.50°/s, was selected for a vestibular implant. Nevertheless, these differences did not reach statistical significance (*p* = 0.329).

**Figure 7:**
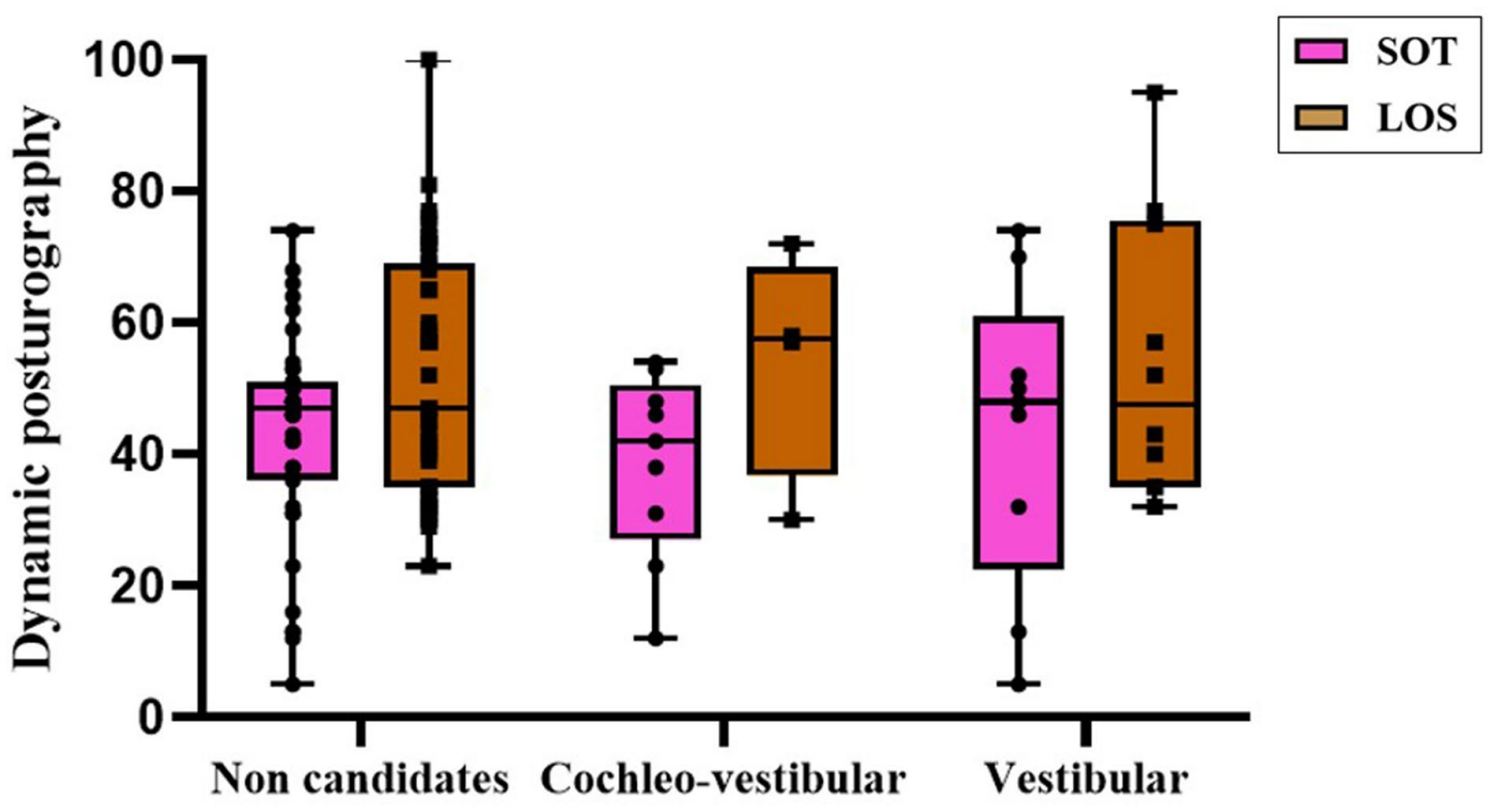
Posturographic performance according to implant candidacy, assessed by SOT and LOS scores. Comparisons were performed between candidacy groups within each posturographic parameter. Boxes represent the median and interquartile range (IQR); whiskers indicate the minimum and maximum values; individual datapoints are shown.

Because absent VEMP responses constituted a prerequisite for implant candidacy, no intergroup comparison of VEMP outcomes was performed in this section, in contrast to Section 3.3, as all implant candidates lacked measurable responses whereas non-candidates did not. Despite the absence of statistically significant differences between groups for posturographic measures (SOT *p* = 0.424; LOS *p* = 0.709), the cochleo-vestibular group tended to exhibit lower SOT scores. Interestingly, the non-candidate group—despite moderate SOT performance—showed the lowest LOS values, suggesting less efficient dynamic postural strategies. These results are detailed in Table 6 and illustrated in Figure 7.

## 4 Discussion

Since the publication and widespread adoption of the Bárány diagnostic criteria for BVP, there has been increasing interest in refining the clinical characterization of this condition, with particular emphasis on better defining its underlying etiologies. Our cohort exhibits an etiological distribution that differs from previous reports, with a markedly lower proportion of idiopathic cases and a higher prevalence of well-defined etiologies. This likely reflects the growing incorporation of advanced diagnostic tools, including high-resolution neuroimaging, genetic testing, and quantitative VOR evaluation, which have enabled the reclassification of many cases in line with actual refinement trend in BVP diagnosis.

Whereas earlier studies reported approximately 50% of BVP cases as idiopathic (1, 3, 4), our cohort shows this figure reduced to around 30%, with an increased representation of cerebellar ataxias and CANVAS related to RFC1 repeat expansions (23). This pattern supports recent hypotheses (5) highlighting the need for routine genetic assessment to reveal causative factors that would otherwise remain unidentified, thereby minimizing the overuse of the idiopathic label. Accurate etiological identification is not only essential from a diagnostic standpoint but also directly impacts therapeutic decision-making and functional prognosis, particularly in the field of vestibular implants. Indeed, previous studies (24) have reported suboptimal outcomes in patients with DFNA9 genetic-related etiologies receiving combined cochleo-vestibular devices, underscoring the importance of etiology-specific candidacy assessment in future implant protocols.

Our results, when compared to other pattern-based classifications, show a similar tendency: across etiologies, the magnitude of VOR loss emerged as the most discriminative functional marker, showing clear separation between idiopathic or Ménière phenotypes and more severe vestibulotoxic or CANVAS-related deficits (Figure 2). This observation aligns with previously published quantitative cluster models (25, 26), in which horizontal and posterior canal weakness represent the primary determinant of functional disability, while additional sensory system involvement influences clinical severity but does not define the phenotype itself. In this context, CANVAS displayed the most profound multicanal impairment in our cohort, reinforcing the notion that these patients cluster at the severe end of the BVP spectrum despite sometimes preserving better hearing thresholds than cochleo-vestibular etiologies (27).

In our sample, we also observed meaningful associations between canal hypofunction and otolith impairment, as reflected by reduced VOR gain and abnormal IAAR values (Table 3). Disease-specific patterns further reinforced this relationship. This constellation of findings suggests a convergent multiaxial deficit affecting both peripheral vestibular sensors and central integrative pathways. Such combined deficits are consistent with previous reports by Tarnutzer et al. (26) and Lempert et al. (28), who demonstrated that overlapping involvement of multiple vestibular end organs is associated with more severe functional deterioration.

From a pathophysiological perspective, the heterogeneous involvement of canals and otolith organs across etiologies likely reflects selective vulnerability of vestibular subsystems. Differences in afferent composition, susceptibility to toxic, inflammatory, genetic, or degenerative processes may explain why certain diseases preferentially affect canal-driven reflexes, otolith-mediated responses, or both. For example, our results show that vestibulotoxic injury has been associated with predominant canal dysfunction with relative otolith preservation, whereas genetic or neurodegenerative conditions such as CANVAS may produce a more diffuse involvement encompassing canals, otoliths, and cerebellar integration. This canal–otolith dissociation, or conversely their coupled impairment, likely contributes to the wide clinical heterogeneity observed in BVP and may underlie differences in symptom expression, compensation capacity, and functional prognosis.

Conversely, a similar correlation was observed at the opposite end of the functional spectrum: idiopathic and Ménière’s disease patients tended to preserve higher canal gains, measurable IAAR responses, and more efficient graviceptive control, supporting a milder and more peripheral phenotype. This parallel distribution across functional axes suggests that SCC–otolith coupling remains symmetrical in both preserved and severely impaired states. Interestingly, however, other etiologies such as vestibulotoxic injury showed a relative dissociation, with profound canal loss but partially preserved otolithic pathways, again in line with previous findings that confirm that disease-specific patterns may reflect differential vulnerability of canal versus otolith organs depending on the underlying pathology (28).

The strong and independent association between reduced LSC and PSC gain and the presence of oscillopsia (Table 4) underscores the role of global VOR insufficiency in dynamic visual stabilization rather than isolated dysfunction of a single semicircular canal. Although oscillopsia has classically been linked to vertical canal impairment, visual instability during daily activities arises from multiaxial head movements in which horizontal components predominate during locomotion. In this context, severe LSC hypofunction may substantially increase retinal slip during walking and other dynamic tasks, thereby contributing directly to the subjective perception of oscillopsia (6, 29).

In parallel, the significant contribution of PR indices—reflecting saccadic timing and phase dispersion—further refines this interpretation, indicating that oscillopsia arises not only from reduced VOR magnitude but also from temporal disorganization of gaze-stabilizing mechanisms, including covert saccades, or the presence of spontaneous nystagmus; however, these variables were not modeled as primary determinants in the present analysis, as their influence has been extensively characterized in prior studies (6, 30). Interestingly, canal-dependent measures of visual stability, such as dynamic visual acuity, and multisensory postural control tests (SOT and LOS) showed weaker or absent correlations with oscillopsia despite clear inter-etiological differences (Tables 2, 3). This dissociation suggests that postural control and visual stability can be independently compromised (31, 32): patients with CANVAS, for example, exhibited severe SOT impairment but oscillopsia rates comparable to vestibulotoxic BVP, while non-candidates for implantation showed paradoxically low LOS values despite better VOR performance. Together, these findings reinforce the concept that postural compensation relies largely on multisensory substitution, whereas oscillopsia remains mechanistically tethered to VOR failure (33, 34)—particularly involving both LSC and PSC function—as also demonstrated by our model.

When considering implant candidacy, the combination of bilateral vestibular hypofunction and non-aidable sensorineural hearing loss with poor speech recognition, becomes a key determinant (5). In our cohort, patients most frequently classified as cochleo-vestibular implant candidates were those with post-infectious etiologies, particularly meningitis-related BVP. This finding is consistent with otopathological evidence of combined cochlear and vestibular injury in *labyrinthitis ossificans* (35), where profound deafness coexists with bilateral vestibular failure, resulting in a homogeneous and well-defined indication for combined stimulation (36).

Another noteworthy finding is that patients with Ménière’s disease were among those most frequently considered candidates for implantation. Although progression to profound deafness is relatively uncommon in Ménière’s disease [approximately 4–6% (37, 38)], cochlear implant eligibility in this population cannot be inferred from pure-tone thresholds alone. Speech understanding is frequently disproportionately impaired relative to PTA, reflecting a well-described tonal–verbal dissociation that may result in non-aidable hearing despite only moderate threshold elevation (39). Moreover, several additional factors increase the likelihood of cochlear implant candidacy in Ménière’s disease, including bilateral involvement, specific disease endotypes and phenotypes (40, 41), long-standing disease duration, and structural cochlear changes such as fibrosis or sequelae following ablative or subablative treatments (42, 43).

Although SSC sparing has been described as a characteristic feature of specific BVP etiologies (26), our results indicate that preservation of SSC function alone does not independently determine implant candidacy. In our cohort, SSC gains were consistently higher than those of the lateral and posterior canals, with significant intergroup differences, as non-candidates exhibited relatively preserved SSC function compared with both vestibular and cochleo-vestibular implant candidates (Table 6), likely reflecting a less advanced stage of vestibular impairment. However, once criteria for severe bilateral vestibular hypofunction were met, residual SSC function did not influence implant allocation, which was primarily driven by horizontal canal dysfunction in accordance with the Bárány Society definition of BVP. However, and strikingly, in a small number of borderline cases, caloric testing provided additional clinically relevant information: two patients with relatively preserved horizontal vHIT gains (>0.6) but markedly reduced caloric responses (SPV 1.10 and 0.50 °/s) were ultimately considered implant candidates. This suggests that, while not a primary determinant, caloric testing may support decision-making in selected cases at the margins of implant eligibility.

Looking forward, the coexistence of different implant modalities offers the opportunity to strategically match each device to the expected pattern of benefit according to the underlying disease mechanism. Patients with disabling oscillopsia are likely to benefit preferentially from vestibular-focused neurostimulation, particularly approaches aimed at restoring the VOR, such as semicircular canal stimulation strategies (44). These approaches have demonstrated partial restoration of both the VOR (45) and the vestibulo-collic reflexes (46, 47) in implanted patients, indicating that gaze stabilization and postural control—core functions of the balance system—can be at least partially re-established through vestibular implantation, thereby contributing to meaningful improvements in quality-of-life outcomes (48). In contrast, etiologies that exhibited a markedly higher burden of postural instability and falls, may benefit more from implant paradigms that emphasize improvements in spatial orientation and graviceptive function through otolithic stimulation (16, 49). These observations support a tailored therapeutic framework in which device selection is driven by the specific neurofunctional profile associated with each etiology. Consequently, the path toward personalized balance restoration will depend on translating detailed vestibular phenotyping into targeted implant indication and outcome prediction. In fact, our group has already demonstrated promising experience in this direction, showing that etiological classification can reliably reflect distinct neurofunctional patterns in BVP (50).

### 4.1 Limitations

This study has several limitations that should be acknowledged. First, its retrospective design may introduce selection and information biases, and causality cannot be directly inferred from the associations reported. Second, although our sample of definite BVP cases was relatively large, several etiologies remained modest in size, reducing the statistical power for subgroup analyses and widening confidence intervals in those categories. Third, we relied on standard clinical metrics for oscillopsia, based on patient-reported experience. Since oscillopsia is intrinsically a dynamic symptom, the lack of treadmill-based measurements or high-velocity gaze-stabilization testing may underestimate its real functional. Even more, the measurement of some vestibular tests as the lack of quantitative caloric SPV values, as responses were analyzed in a binary manner based on the presence or absence of nystagmus during ice-water stimulation, which does not allow strict application of the Bárány bithermal SPV criterion. Finally, the cross-sectional nature of our analysis does not allow evaluation of longitudinal changes in compensation or auditory–vestibular evolution, which may influence implant candidacy over time.

## 5 Conclusion

This study provides new evidence that BVP is not a single disorder but a collection of etiologically driven phenotypes with distinct implications for symptom burden and therapeutic decision-making. By demonstrating that symptoms as oscillopsia is mostly determined by the degree VOR loss—and not by otolith function or graviceptive decline—we identify a specific physiological target for future vestibular restoration strategies. Moreover, the marked heterogeneity in auditory and vestibular involvement across etiologies supports a shift from uniform implant indications toward precision vestibular medicine, in which device selection (vestibular versus cochleo-vestibular implantation) is tailored to an individual’s neurofunctional profile. Prospective, dynamic assessments and post-implant outcomes will be critical next steps to operationalize this personalized approach and ultimately improve real-world balance and vision in patients with BVP.

## Data Availability

The original contributions presented in the study are included in the article/Supplementary material, further inquiries can be directed to the corresponding author.

## References

[1] Strupp M Kim JS Murofushi T Straumann D Jen JC Rosengren SM . Bilateral vestibulopathy: diagnostic criteria consensus document of the classification committee of the Bárány society. J Vestib Res. (2017) 27:177–89. doi: 10.3233/VES170619 29081426 PMC9249284

[2] Herssens N How D van de Berg R McCrum C. Falls among people with bilateral vestibulopathy: a review of causes, incidence, injuries, and methods. JAMA Otolaryngol Head Neck Surg. (2022) 148:187–92. doi: 10.1001/jamaoto.2021.3673, 34989780

[3] Lucieer F Vonk P Guinand N Stokroos R Kingma H van de Berg R. Bilateral vestibular hypofunction: insights in etiologies, clinical subtypes, and diagnostics. Front Neurol. (2016) 7:26. doi: 10.3389/fneur.2016.00026, 26973594 PMC4777732

[4] Pérez-Fernández N Alvarez-Gomez L Manrique-Huarte R. Bilateral vestibular hypofunction in the time of the video head impulse test. Audiol Neurotol. (2019) 25:72–8. doi: 10.1159/000504286, 31825921

[5] Moyaert J Dobbels B Peetermans O Boon B Lucieer F Guinand N . Etiologies and hearing status in bilateral vestibulopathy: a retrospective study of 315 patients. Front Neurol. (2023) 14:1271012. doi: 10.3389/fneur.2023.1271012, 38093757 PMC10716460

[6] Batuecas-Caletrio A Trinidad-Ruiz G Rey-Martinez J Matiño-Soler E Martin Sanz E Perez FN. Oscillopsia in bilateral vestibular hypofunction: not only gain but saccades too. Ear Hear. (2020) 41:323–9. doi: 10.1097/AUD.0000000000000760, 31517671

[7] Harrell RG Cassidy AR Klatt BN Hovareshti P Whitney SL. Vestibular rehabilitation in cerebellar ataxia with neuropathy and vestibular areflexia syndrome (CANVAS)—a case report. J Otol. (2023) 18:199–207. doi: 10.1016/j.joto.2023.06.004, 37877066 PMC10593570

[8] Starkov D Strupp M Pleshkov M Kingma H van de Berg R. Diagnosing vestibular hypofunction: an update. J Neurol. (2020) 268:377–85. doi: 10.1007/s00415-020-10139-4, 32767115 PMC7815536

[9] van de Berg R Guinand N Stokroos RJ Guyot JP Kingma H. The vestibular implant: quo vadis? Front Neurol. (2011) 2:47. doi: 10.3389/fneur.2011.00047, 21991260 PMC3181464

[10] Lacour M Helmchen C Vidal PP. Vestibular compensation: the neuro-otologist’s best friend. J Neurol. (2016) 263:S54–64. doi: 10.1007/s00415-015-7903-4 27083885 PMC4833803

[11] Lacour M Bernard-Demanze L. Interaction between vestibular compensation mechanisms and vestibular rehabilitation therapy: 10 recommendations for optimal functional recovery. Front Neurol. (2015) 5:285. doi: 10.3389/fneur.2014.00285, 25610424 PMC4285093

[12] Hall CD Herdman SJ Whitney SL Cass SP Clendaniel RA Fife TD . Vestibular rehabilitation for peripheral vestibular hypofunction: an evidence-based clinical practice guideline: from the American Physical Therapy Association Neurology Section. J Neurol Phys Ther. (2016) 40:124–55. doi: 10.1097/NPT.0000000000000120, 26913496 PMC4795094

[13] Flix-Díez L Blanco-Pareja M Pérez-Fernández N. Limits of stability during a therapeutic exercise intervention for instability: progression, responders’ and non-responders’ analysis and predictors. J Clin Med. (2024) 13:5036. doi: 10.3390/jcm13175036, 39274248 PMC11396621

[14] Pérez-Fornos A Guinand N van de Berg R Stokroos R Micera S Kingma H . Artificial balance: restoration of the vestibulo-ocular reflex in humans with a prototype vestibular implant. Front Neurol. (2014) 5:66. doi: 10.3389/fneur.2014.00066 24808890 PMC4010770

[15] Della Santina CC Migliaccio AA Hayden R Melvin TA Fridman GY Chiang B . Current and future management of bilateral loss of vestibular sensation—an update on the Johns Hopkins multichannel vestibular prosthesis project. Cochlear Implants Int. (2010) 11:2–11. doi: 10.1179/146701010X12726366068454, 21756683 PMC3270064

[16] de Ramos Miguel A Sluydts M Falcón JC Manrique-Huarte R Rodríguez L Zarowski A . Enhancing balance and auditory function in bilateral vestibulopathy through otolithic vestibular stimulation: insights from a pilot study on cochlea-vestibular implant efficacy. Front Neurol. (2025) 16:1520554. doi: 10.3389/fneur.2025.1520554 39949795 PMC11821918

[17] de Ramos Miguel A Rodríguez-Montesdeoca I Falcón-González JC Borkoski-Barreiro S Zarowski A Sluydts M . Stimulation crosstalk between cochlear and vestibular 257 spaces during cochlear electrical stimulation. Laryngoscope. (2024) 134:2349–55. doi: 10.1002/lary.31174 38010817

[18] Rey-Martinez J Batuecas-Caletrio A Matino E Perez Fernandez N. HITCal: a software tool for analysis of video head impulse test responses. Acta Otolaryngol. (2015) 135:886–94. doi: 10.3109/00016489.2015.1035401, 25857220

[19] Guajardo-Vergara C Pérez-Fernandez N. Air and bone stimulation in vestibular evoked myogenic potentials in patients with unilateral Ménière’s disease and in controls. Hear Balance Commun. (2019) 17:170–8. doi: 10.1080/21695717.2019.1591009

[20] Cevette MJ Puetz B Marion MS Wertz ML Muenter MD. Aphysiologic performance on dynamic posturography. Otolaryngol Head Neck Surg. (1995) 112:676–88. doi: 10.1016/s0194-59989570175-3, 7777351

[21] Strupp M Magnusson M. Acute unilateral vestibulopathy. Neurol Clin. (2015) 33:669–85. doi: 10.1016/j.ncl.2015.04.012, 26231279

[22] Cárdenas MR Marrero-Aguiar V. Cuaderno de Logoaudiometría. Madrid, Spain: UNED (1994) ISBN 84-362-3009-4.

[23] Szymanska Heydel M Heindl F Hartmann A Borsche M Traschütz A Straumann D . The spectrum of peripheral-vestibular deficits and their change over time in CANVAS/RFC1-related ataxia systematic review and meta-analysis of quantitative head-impulse testing. Cerebellum. (2025) 24:67. doi: 10.1007/s12311-025-01825-y 40111638 PMC11926034

[24] Guinand N van de Berg R Ranieri M Cavuscens S DiGiovanna J Nguyen TAK . Vestibular implants: hope for improving the quality of life of patients with bilateral vestibular loss. Annu Int Conf IEEE Eng Med Biol Soc. (2015) 2015:7192–5. doi: 10.1109/EMBC.2015.7320051 26737951

[25] Tarnutzer AA Bockisch CJ Buffone E Weiler S Bachmann LM Weber KP. Disease-specific sparing of the anterior semicircular canals in bilateral vestibulopathy. Clin Neurophysiol. (2016) 127:2791–801. doi: 10.1016/j.clinph.2016.05.005, 27417055

[26] Tarnutzer AA Bockisch CJ Buffone E Weber KP. Hierarchical cluster analysis of semicircular canal and otolith deficits in bilateral vestibulopathy. Front Neurol. (2018) 9:9. doi: 10.3389/fneur.2018.00244, 29692756 PMC5902493

[27] Garces P Antoniades CA Sobanska A Kovacs N Ying SH Gupta AS . Quantitative oculomotor assessment in hereditary ataxia: discriminatory power, correlation with severity measures, and recommended parameters for specific genotypes. Cerebellum. (2024) 23:121–35. doi: 10.1007/s12311-023-01514-8, 36640220 PMC10864420

[28] Lempert T Gianna CC Gresty MA Bronstein AM. Effect of otolith dysfunction. Impairment of visual acuity during linear head motion in labyrinthine defective subjects. Brain. (1997) 120:1005–13. doi: 10.1093/brain/120.6.1005 9217684

[29] Agrawal Y Bremova T Kremmyda O Strupp M. Semicircular canal, saccular and utricular function in patients with bilateral vestibulopathy: analysis based on etiology. J Neurol. (2013) 260:876–83. doi: 10.1007/s00415-012-6724-y, 23104126 PMC4069122

[30] Straube A Bronstein A Straumann D. Nystagmus and oscillopsia. Eur J Neurol. (2011) 19:6–14. doi: 10.1111/j.1468-1331.2011.03503.x, 21906211

[31] Herssens N Saeys W Vereeck L Meijer K van de Berg R Van Rompaey V . An exploratory investigation on spatiotemporal parameters, margins of stability, and their interaction in bilateral vestibulopathy. Sci Rep. (2021) 11:6427. doi: 10.1038/s41598-021-85870-7, 33742071 PMC7979710

[32] Boutabla A Revol R Carvalho MF Grouvel G Corre J Cugnot JF . Gait impairments in patients with bilateral vestibulopathy and chronic unilateral vestibulopathy. Front Neurol. (2025) 16:1547444. doi: 10.3389/fneur.2025.1547444, 40083452 PMC11903280

[33] Batuecas-Caletrio A Rey-Martinez J Trinidad-Ruiz G Matiño-Soler E Pérez-Fernández N . Vestibulo-ocular reflex stabilization after vestibular schwannoma surgery: a story told by saccades. Front Neurol. (2017) 8:15. doi: 10.3389/fneur.2017.00015 28179894 PMC5263125

[34] Trinidad-Ruiz G Rey-Martinez J Batuecas-Caletrio A Matiño-Soler E Perez-Fernandez N. Visual performance and perception as a target of saccadic strategies in patients with unilateral vestibular loss. Ear Hear. (2018) 39:1176–86. doi: 10.1097/AUD.0000000000000576, 29578887

[35] West N Sass H Klokker M Cayé-Thomasen P. Functional loss after meningitis—evaluation of vestibular function in patients with postmeningitic hearing loss. Front Neurol. (2020) 11:11. doi: 10.3389/fneur.2020.00681, 32849181 PMC7406674

[36] van de Berg R Van Tilburg M Kingma H. Bilateral vestibular hypofunction: challenges in establishing the diagnosis in adults. ORL J Otorhinolaryngol Relat Spec. (2015) 77:197–218. doi: 10.1159/000433549, 26366566

[37] Mick P Amoodi H Arnoldner C Shipp D Friesen L Lin V . Cochlear implantation in patients with advanced Ménière’s disease. Otol Neurotol. (2014) 35:1172–8. doi: 10.1097/mao.0000000000000202, 24366468

[38] Prenzler NK Bültmann E Giourgas A Steffens M Salcher RB Stolle S . Cochlear implantation in patients with definite Meniere’s disease. Eur Arch Otorrinolaringol. (2017) 274:751–6. doi: 10.1007/s00405-016-4356-z, 27783138

[39] Lorente-Piera J Blanco M Santos-Garrido J Manrique-Huarte R Suárez-Vega V Domínguez P . Functional audiometric dissociation in Ménière’s disease: exploring the mismatch between pure-tone thresholds and speech recognition. J Clin Med. (2025) 14:4747. doi: 10.3390/jcm14134747 40649120 PMC12250289

[40] Chien C Kulthaveesup A Herrmann BS Rauch SD. Cochlear implantation hearing outcome in Ménière’s disease. Otolaryngol Head Neck Surg. (2021) 166:523–9. doi: 10.1177/01945998211012298 34003698

[41] Brühlmann C Spiegel JL Mühle A Dalbert A Lin VY Le TN . Deafness progressing to cochlear implant eligibility is eight times more likely in the hypoplastic than the degenerative endotype of Menière’s disease. Otol Neurotol. (2025) 46:e170–5. doi: 10.1097/MAO.0000000000004482 40164978 PMC12105948

[42] Selleck AM Dillon M Perkins E Brown KD. Cochlear implantation in the setting of Menière’s disease after Labyrinthectomy: a Meta-analysis. Otol Neurotol. (2021) 42:e973–9. doi: 10.1097/MAO.0000000000003200, 34049331

[43] Mukherjee P Eykamp K Brown D Curthoys I Flanagan S Biggs N . Cochlear implantation in Meniere’s disease with and without labyrinthectomy. Otol Neurotol. (2017) 38:192–8. doi: 10.1097/MAO.0000000000001278 27861194

[44] van de Berg R. The vestibular implant: feasibility in humans (doctoral thesis). Maastricht University, Maastricht, 224 (2018)

[45] Perez Fornos A Cavuscens S Ranieri M van de Berg R Stokroos R Kingma H . The vestibular implant: a probe in orbit around the human balance system. J Vestib Res. (2017) 27:51–61. doi: 10.3233/VES-170604, 28387690

[46] van de Berg R Guinand N Nguyen TAK Ranieri M Cavuscens S Guyot JP . The vestibular implant: frequency-dependency of the electrically evoked vestibulo-ocular reflex in humans. Front Syst Neurosci. (2015) 8:255. doi: 10.3389/fnsys.2014.00255 25653601 PMC4299437

[47] van Boxel SCJ Vermorken BL Volpe B Guinand N Perez-Fornos A Devocht EMJ . The vestibular implant: effects of stimulation parameters on the electrically-evoked vestibulo-ocular reflex. Front Neurol. (2024) 15:1483067. doi: 10.3389/fneur.2024.1483067 39574507 PMC11579865

[48] Chow MR Ayiotis AI Schoo DP Gimmon Y Lane KE Morris BJ . Posture, gait, quality of life, and hearing with a vestibular implant. N Engl J Med. (2021) 384:521–32. doi: 10.1056/NEJMoa2020457, 33567192 PMC8477665

[49] Miwa T Kouga T Ito T Tarui A Asai Y Fujikawa T . Development of a vestibular implant for otolith stimulation: a mouse model study. Auris Nasus Larynx. (2025) 52:412–9. doi: 10.1016/j.anl.2025.06.007, 40543096

[50] Lorente-Piera J Prieto E Ramos de Miguel A Manrique M Pérez-Fernández N Macías ÁR . Clinical research on positron emission tomography imaging of the neuro-stimulation system in patients with Cochleo-vestibular implants: is there a response beyond the peripheral organ? Journal of. Clin Med. (2025) 14:1445. doi: 10.3390/jcm14051445, 40094915 PMC11900547

