# Supplementary material for: Decoding the bilateral vestibulopathy spectrum: etiology-based phenotypes, clinical profiles and pathways to implant candidacy
Source: Front Neurol. 2026 Jan 16;17:1745221. doi: 10.3389/fneur.2026.1745221 (PMC12855053; doi:10.3389/fneur.2026.1745221)
Supplement: Supplementary file 1 [file Data_Sheet_1.pdf]

Audiovestibular Test					p value
**Auditory**	**PTA**	Right ear	60.09±28.86 dB		0.660
		Left Ear	55.83± 30.47 dB		
**Vestibular**	**vHIT (Gain/PR)**	Right SSC	0.52± 0.21		0.415
		Left SSC	0.51± 0.23		
		Right LSC	0.42± 0.17	50.11 [31.25-73.25]	0.713 0.263
		Left LSC	0.38± 0.19	54.53 [32.28-89.00]	
		Right PSC	0.40± 0.18		0.398
		Left PSC	0.38±0.22		
	**Caloric test**	SPV	0.42 ± 0.54 º/s		0.632
	**VEMPS**	cVEMP	16.66 ± 11.72%		0.480
		oVEMP	18.69 ± 13.15%		
	**Dynamic posturography**	SOT	45.35± 17.22		-
		LOS	52.79± 21.40		
	**Visual Acuity**		55.58± 29.33		

Supplementary Table: Summary of the general audiovestibular results in the BVP cohort
